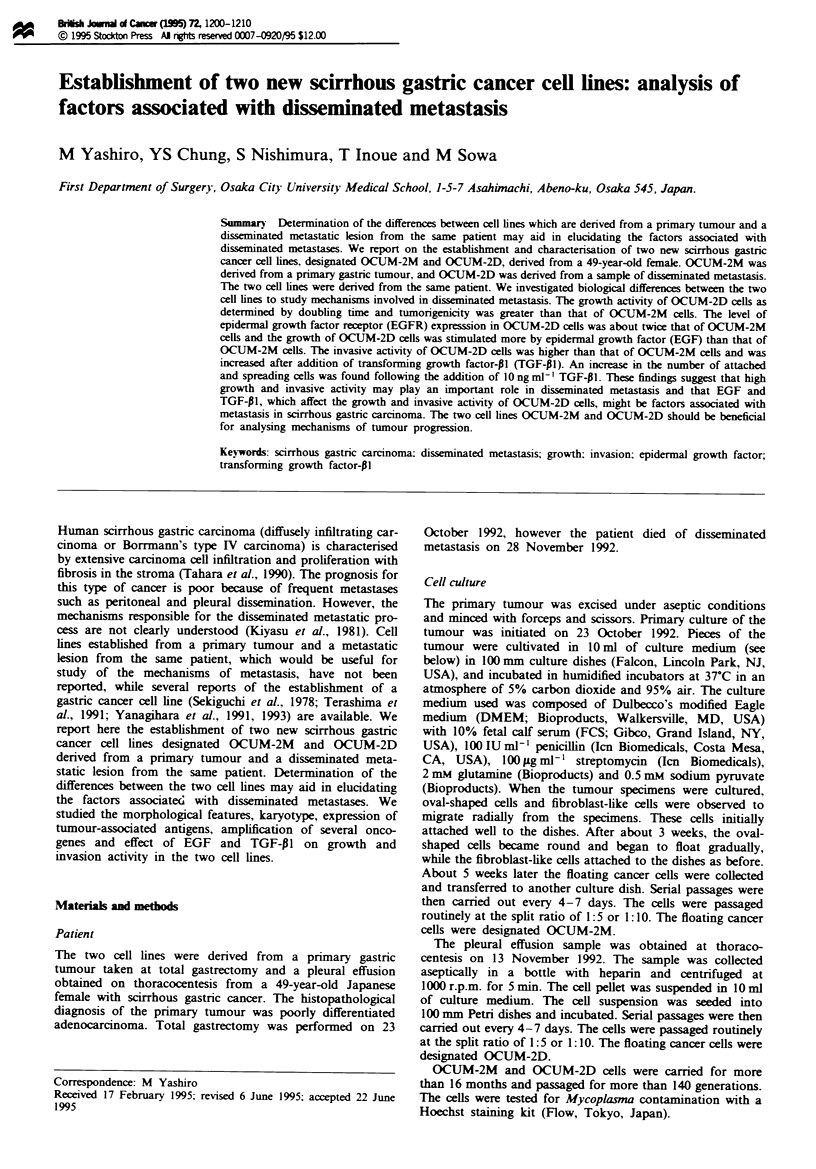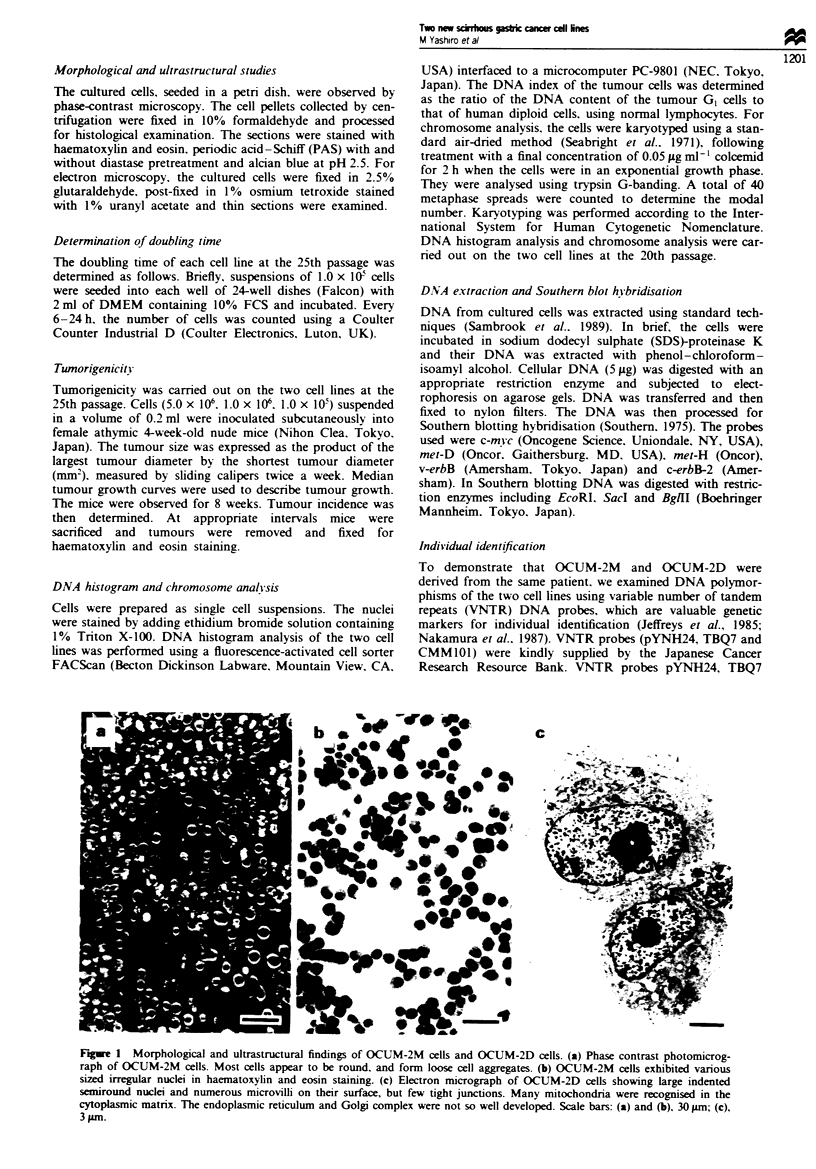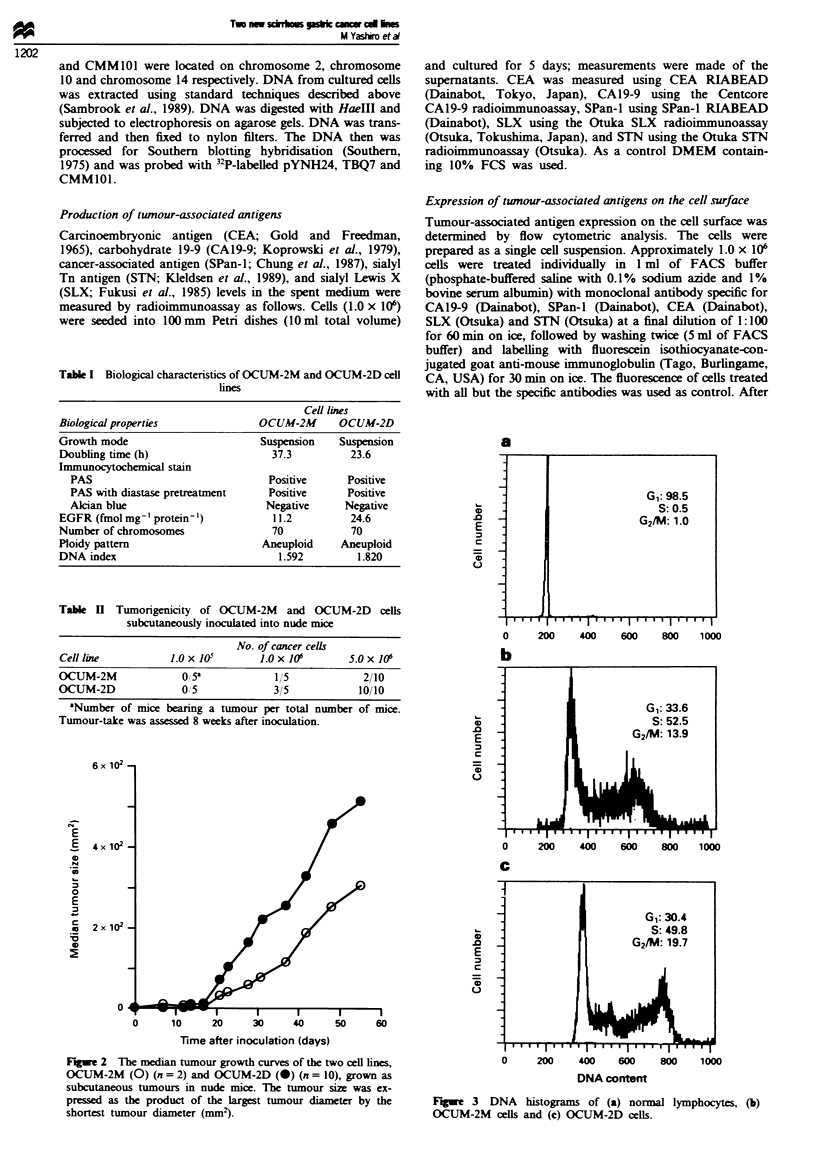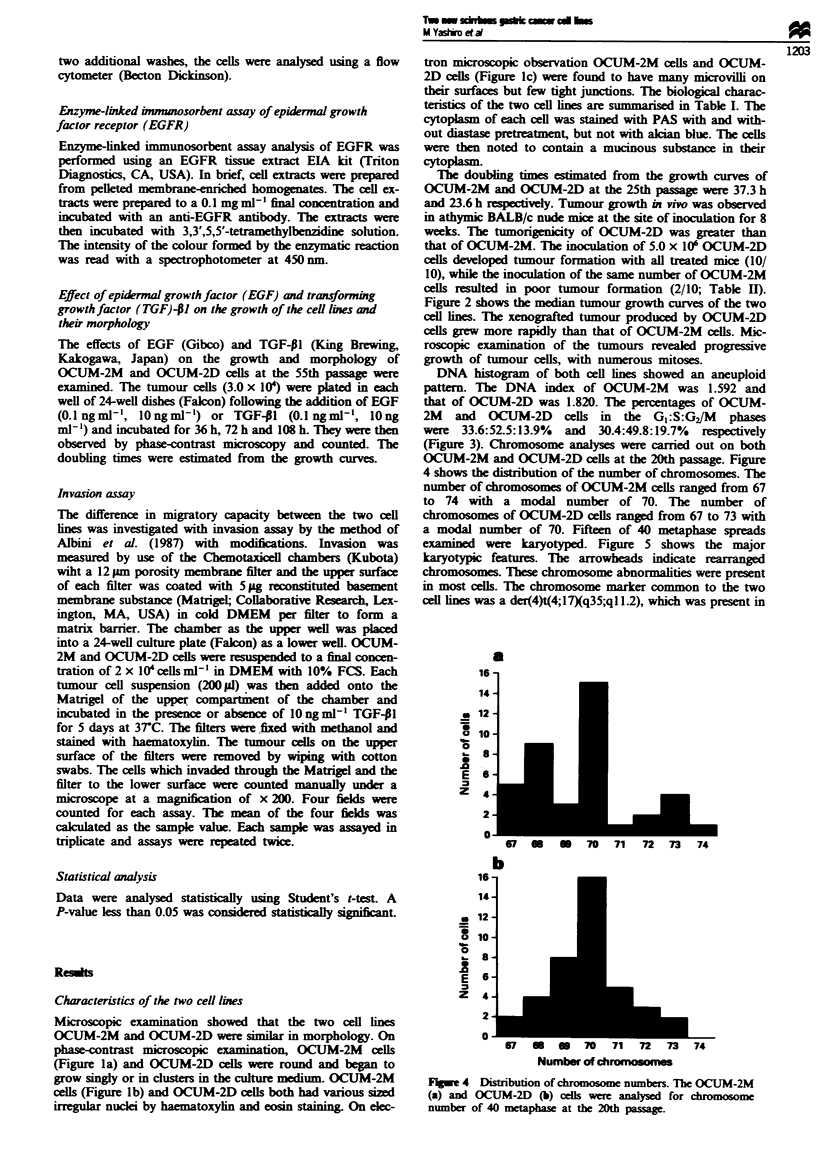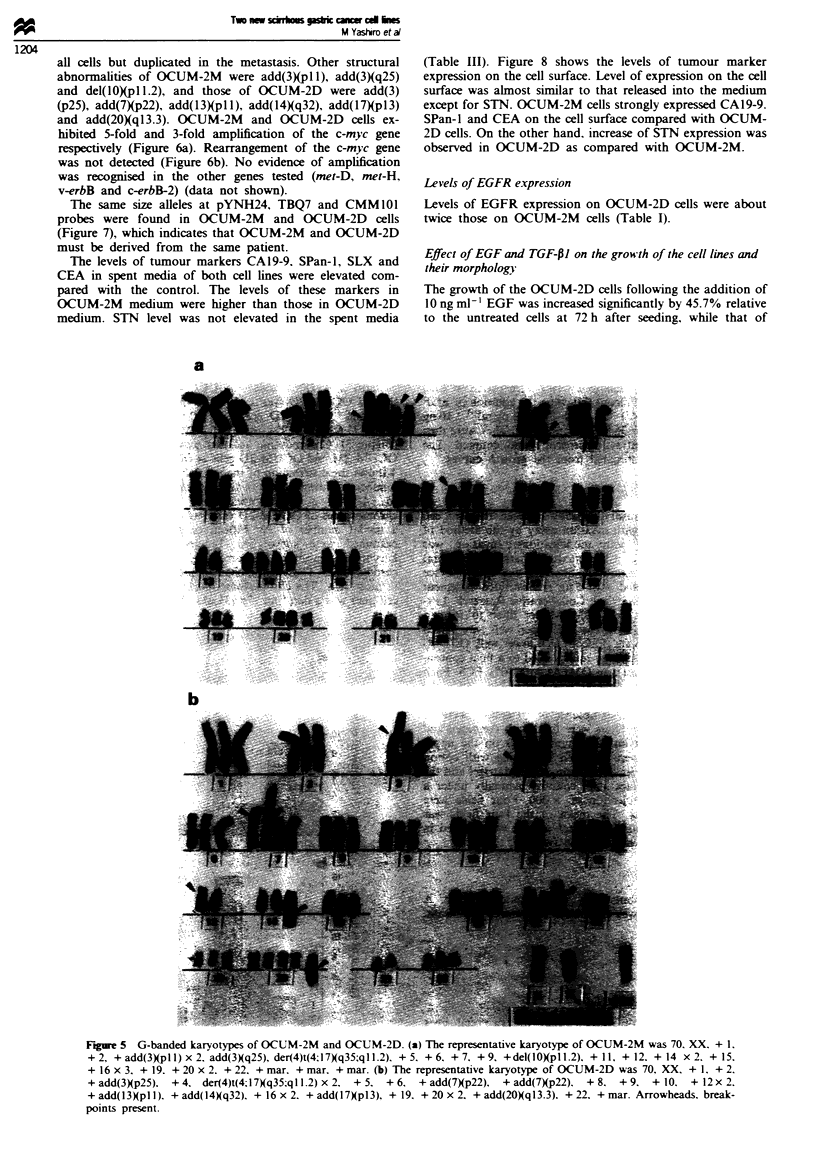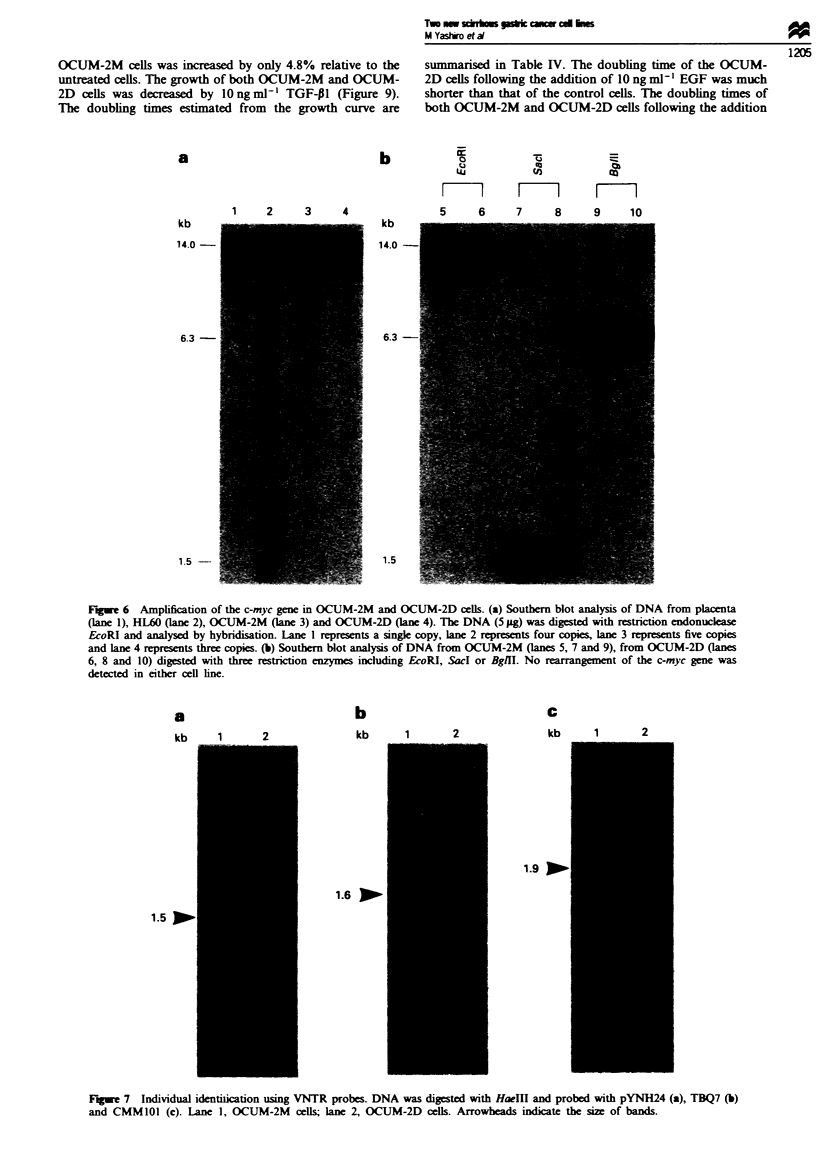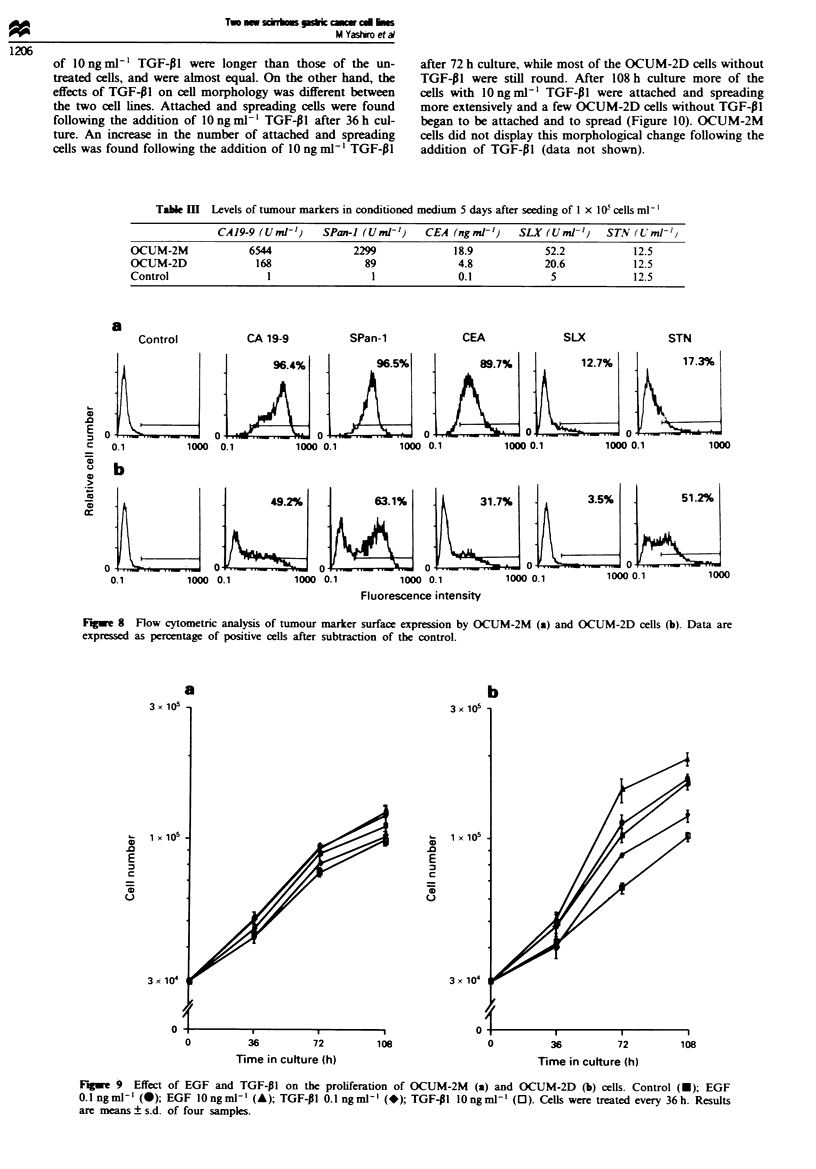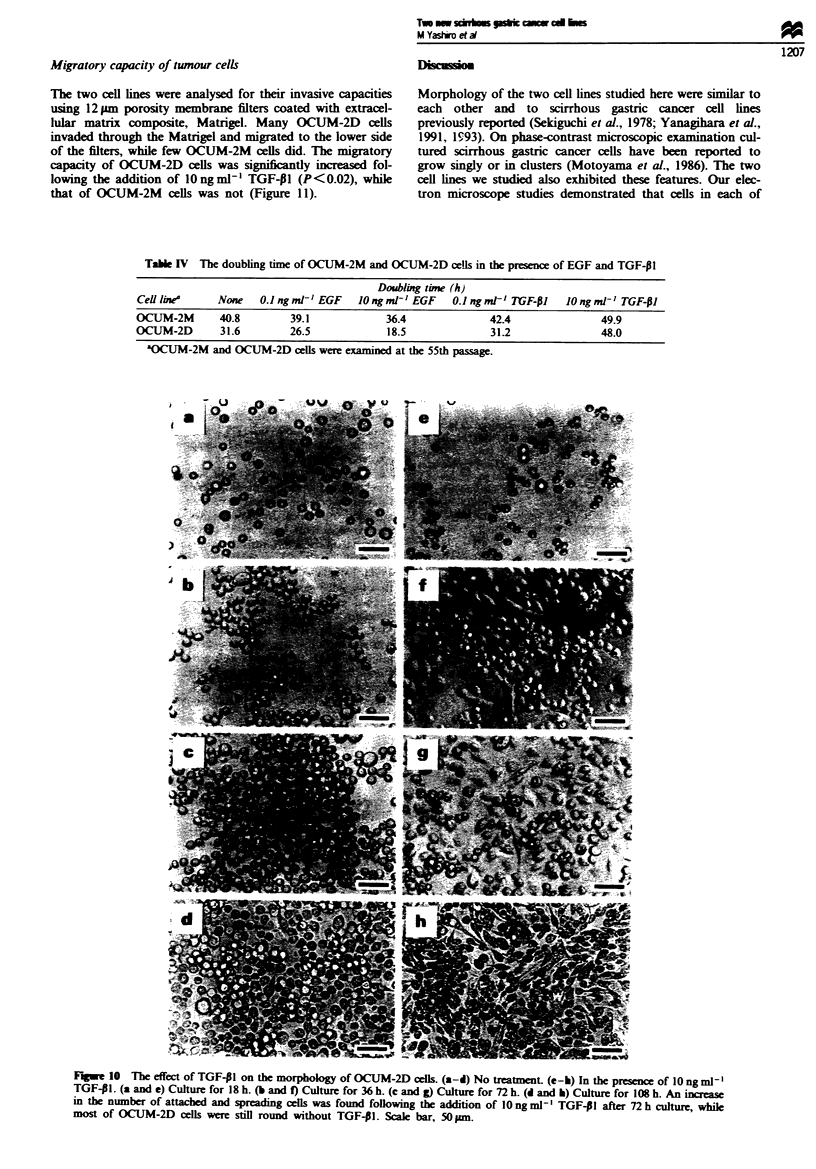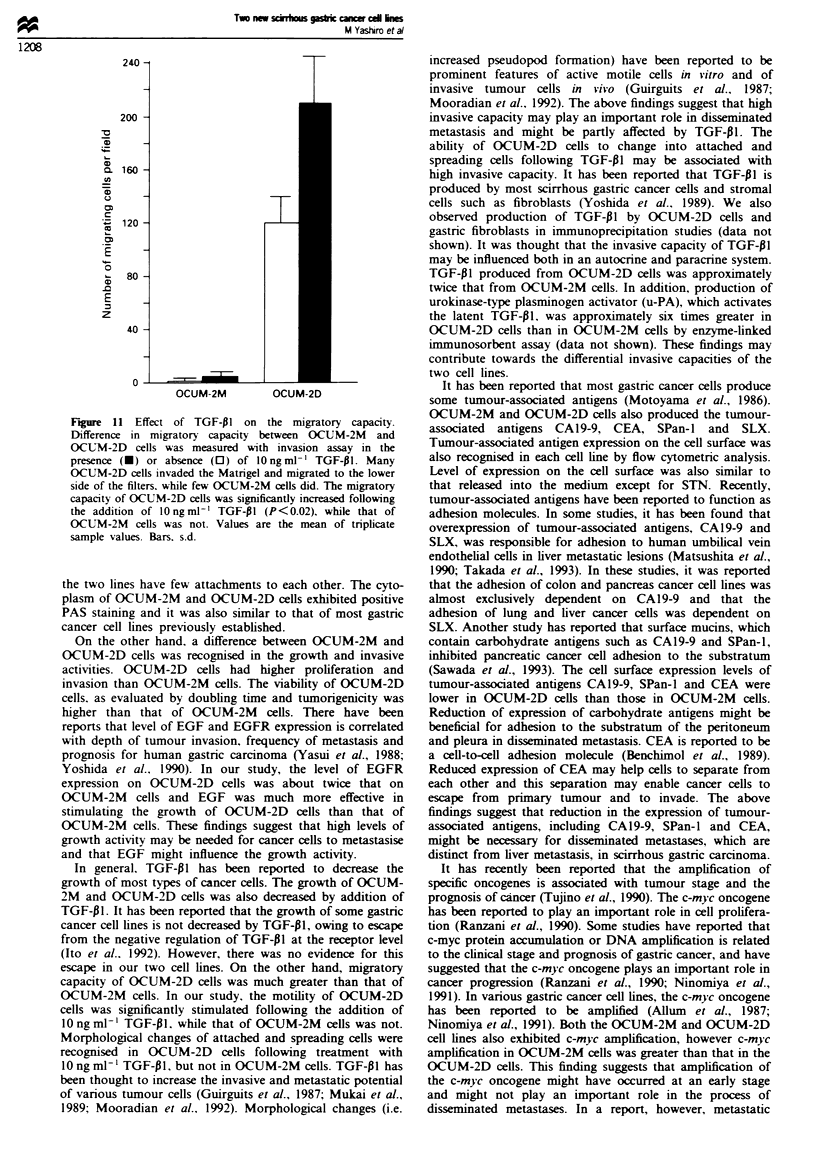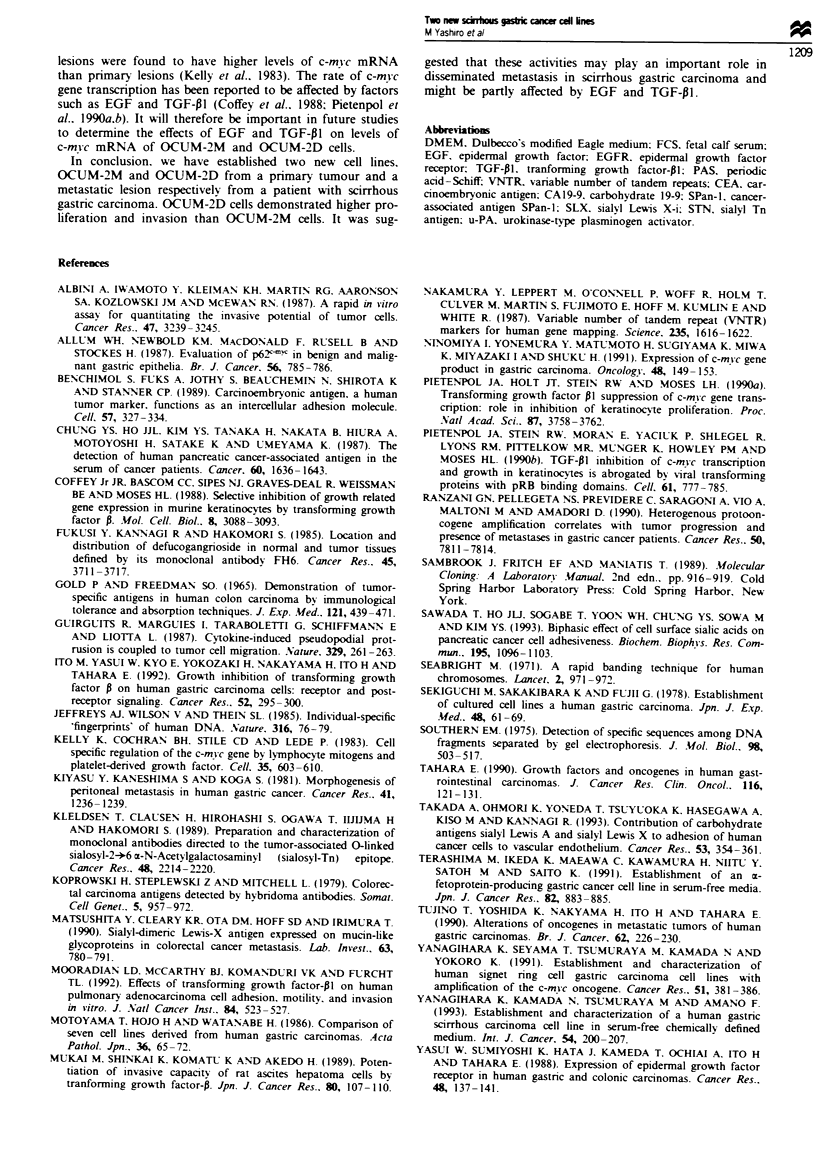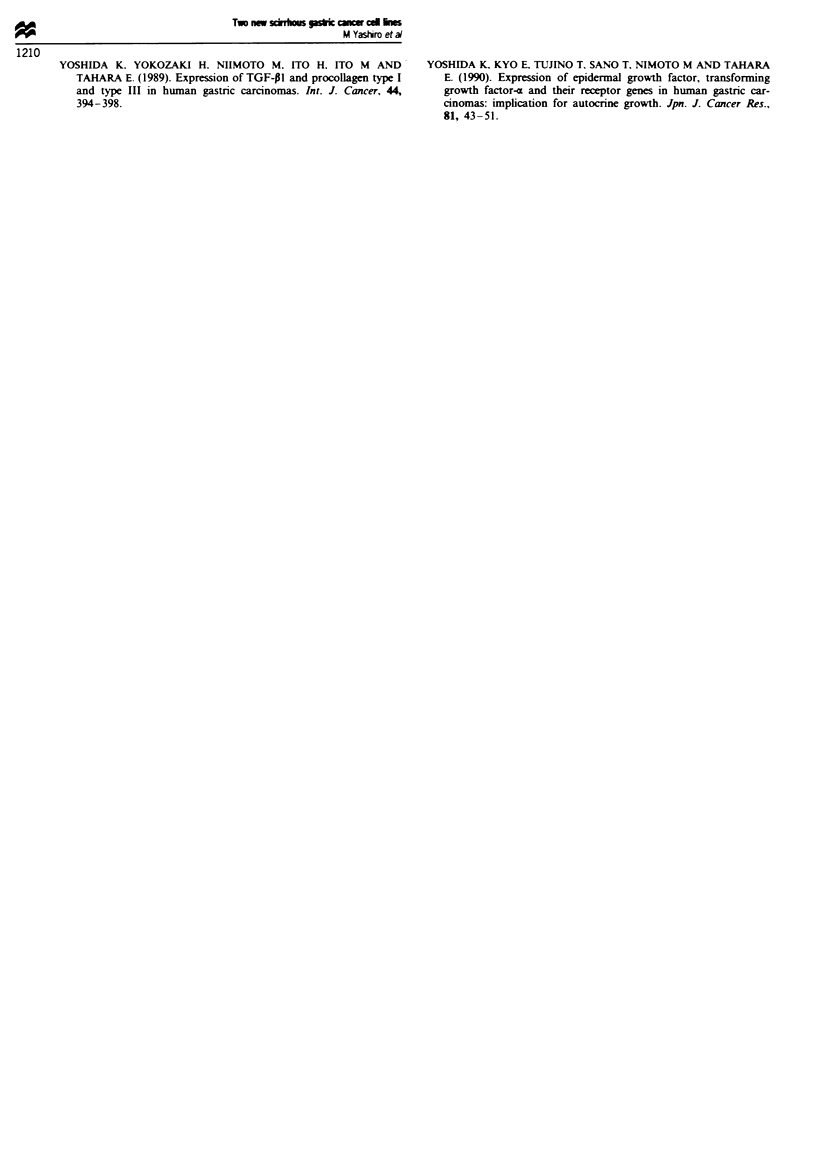# Establishment of two new scirrhous gastric cancer cell lines: analysis of factors associated with disseminated metastasis.

**DOI:** 10.1038/bjc.1995.486

**Published:** 1995-11

**Authors:** M. Yashiro, Y. S. Chung, S. Nishimura, T. Inoue, M. Sowa

**Affiliations:** First Department of Surgery, Osaka City University Medical School, Japan.

## Abstract

**Images:**


British Journal of Cancer (1995) 72, 1200–1210

© 1995 Stockton Press All rights reserved 0007-0920/95 $12.00

### Establishment of two new scirrhous gastric cancer cell lines: analysis of factors associated with disseminated metastasis

M Yashiro, YS Chung, S Nishimura, T Inoue and M Sowa

*First Department of Surgery, Osaka City University Medical School, 1-5-7 Asahimachi, Abeno-ku, Osaka 545, Japan.*

**Summary** Determination of the differences between cell lines which are derived from a primary tumour and a disseminated metastatic lesion from the same patient may aid in elucidating the factors associated with disseminated metastases. We report on the establishment and characterisation of two new scirrhous gastric cancer cell lines, designated OCUM-2M and OCUM-2D, derived from a 49-year-old female. OCUM-2M was derived from a primary gastric tumour, and OCUM-2D was derived from a sample of disseminated metastasis. The two cell lines were derived from the same patient. We investigated biological differences between the two cell lines to study mechanisms involved in disseminated metastasis. The growth activity of OCUM-2D cells as determined by doubling time and tumorigenicity was greater than that of OCUM-2M cells. The level of epidermal growth factor receptor (EGFR) expresssion in OCUM-2D cells was about twice that of OCUM-2M cells and the growth of OCUM-2D cells was stimulated more by epidermal growth factor (EGF) than that of OCUM-2M cells. The invasive activity of OCUM-2D cells was higher than that of OCUM-2M cells and was increased after addition of transforming growth factor-$\beta 1$ (TGF-$\beta 1$). An increase in the number of attached and spreading cells was found following the addition of 10 ng $ml^{-1}$ TGF-$\beta 1$. These findings suggest that high growth and invasive activity may play an important role in disseminated metastasis and that EGF and TGF-$\beta 1$, which affect the growth and invasive activity of OCUM-2D cells, might be factors associated with metastasis in scirrhous gastric carcinoma. The two cell lines OCUM-2M and OCUM-2D should be beneficial for analysing mechanisms of tumour progression.

**Keywords**: scirrhous gastric carcinoma; disseminated metastasis; growth; invasion; epidermal growth factor; transforming growth factor-$\beta 1$

Human scirrhous gastric carcinoma (diffusely infiltrating carcinoma or Borrmann's type IV carcinoma) is characterised by extensive carcinoma cell infiltration and proliferation with fibrosis in the stroma (Tahara *et al.*, 1990). The prognosis for this type of cancer is poor because of frequent metastases such as peritoneal and pleural dissemination. However, the mechanisms responsible for the disseminated metastatic process are not clearly understood (Kiyasu *et al.*, 1981). Cell lines established from a primary tumour and a metastatic lesion from the same patient, which would be useful for study of the mechanisms of metastasis, have not been reported, while several reports of the establishment of a gastric cancer cell line (Sekiguchi *et al.*, 1978; Terashima *et al.*, 1991; Yanagihara *et al.*, 1991, 1993) are available. We report here the establishment of two new scirrhous gastric cancer cell lines designated OCUM-2M and OCUM-2D derived from a primary tumour and a disseminated metastatic lesion from the same patient. Determination of the differences between the two cell lines may aid in elucidating the factors associated with disseminated metastases. We studied the morphological features, karyotype, expression of tumour-associated antigens, amplification of several oncogenes and effect of EGF and TGF-$\beta 1$ on growth and invasion activity in the two cell lines.

### Materials and methods

### *Patient*

The two cell lines were derived from a primary gastric tumour taken at total gastrectomy and a pleural effusion obtained on thoracocentesis from a 49-year-old Japanese female with scirrhous gastric cancer. The histopathological diagnosis of the primary tumour was poorly differentiated adenocarcinoma. Total gastrectomy was performed on 23 October 1992, however the patient died of disseminated metastasis on 28 November 1992.

### *Cell culture*

The primary tumour was excised under aseptic conditions and minced with forceps and scissors. Primary culture of the tumour was initiated on 23 October 1992. Pieces of the tumour were cultivated in 10 ml of culture medium (see below) in 100 mm culture dishes (Falcon, Lincoln Park, NJ, USA), and incubated in humidified incubators at 37°C in an atmosphere of 5% carbon dioxide and 95% air. The culture medium used was composed of Dulbecco's modified Eagle medium (DMEM; Bioproducts, Walkersville, MD, USA) with 10% fetal calf serum (FCS; Gibco, Grand Island, NY, USA), 100 IU $ml^{-1}$ penicillin (Icn Biomedicals, Costa Mesa, CA, USA), 100 $\mu g$ $ml^{-1}$ streptomycin (Icn Biomedicals), 2 mM glutamine (Bioproducts) and 0.5 mM sodium pyruvate (Bioproducts). When the tumour specimens were cultured, oval-shaped cells and fibroblast-like cells were observed to migrate radially from the specimens. These cells initially attached well to the dishes. After about 3 weeks, the oval-shaped cells became round and began to float gradually, while the fibroblast-like cells attached to the dishes as before. About 5 weeks later the floating cancer cells were collected and transferred to another culture dish. Serial passages were then carried out every 4–7 days. The cells were passaged routinely at the split ratio of 1:5 or 1:10. The floating cancer cells were designated OCUM-2M.

The pleural effusion sample was obtained at thoracocentesis on 13 November 1992. The sample was collected aseptically in a bottle with heparin and centrifuged at 1000 r.p.m. for 5 min. The cell pellet was suspended in 10 ml of culture medium. The cell suspension was seeded into 100 mm Petri dishes and incubated. Serial passages were then carried out every 4–7 days. The cells were passaged routinely at the split ratio of 1:5 or 1:10. The floating cancer cells were designated OCUM-2D.

OCUM-2M and OCUM-2D cells were carried for more than 16 months and passaged for more than 140 generations. The cells were tested for *Mycoplasma* contamination with a Hoechst staining kit (Flow, Tokyo, Japan).

Correspondence: M Yashiro
Received 17 February 1995; revised 6 June 1995; accepted 22 June 1995

*Morphological and ultrastructural studies*

The cultured cells, seeded in a petri dish, were observed by phase-contrast microscopy. The cell pellets collected by centrifugation were fixed in 10% formaldehyde and processed for histological examination. The sections were stained with haematoxylin and eosin, periodic acid-Schiff (PAS) with and without diastase pretreatment and alcian blue at pH 2.5. For electron microscopy, the cultured cells were fixed in 2.5% glutaraldehyde, post-fixed in 1% osmium tetroxide stained with 1% uranyl acetate and thin sections were examined.

*Determination of doubling time*

The doubling time of each cell line at the 25th passage was determined as follows. Briefly, suspensions of $1.0 \times 10^5$ cells were seeded into each well of 24-well dishes (Falcon) with 2 ml of DMEM containing 10% FCS and incubated. Every 6–24 h, the number of cells was counted using a Coulter Counter Industrial D (Coulter Electronics, Luton, UK).

*Tumorigenicity*

Tumorigenicity was carried out on the two cell lines at the 25th passage. Cells ($5.0 \times 10^6$, $1.0 \times 10^6$, $1.0 \times 10^5$) suspended in a volume of 0.2 ml were inoculated subcutaneously into female athymic 4-week-old nude mice (Nihon Clea, Tokyo, Japan). The tumour size was expressed as the product of the largest tumour diameter by the shortest tumour diameter ($mm^2$), measured by sliding calipers twice a week. Median tumour growth curves were used to describe tumour growth. The mice were observed for 8 weeks. Tumour incidence was then determined. At appropriate intervals mice were sacrificed and tumours were removed and fixed for haematoxylin and eosin staining.

*DNA histogram and chromosome analysis*

Cells were prepared as single cell suspensions. The nuclei were stained by adding ethidium bromide solution containing 1% Triton X-100. DNA histogram analysis of the two cell lines was performed using a fluorescence-activated cell sorter FACScan (Becton Dickinson Labware, Mountain View, CA, USA) interfaced to a microcomputer PC-9801 (NEC, Tokyo, Japan). The DNA index of the tumour cells was determined as the ratio of the DNA content of the tumour $G_1$ cells to that of human diploid cells, using normal lymphocytes. For chromosome analysis, the cells were karyotyped using a standard air-dried method (Seabright *et al*., 1971), following treatment with a final concentration of 0.05 $\mu g\ ml^{-1}$ colcemid for 2 h when the cells were in an exponential growth phase. They were analysed using trypsin G-banding. A total of 40 metaphase spreads were counted to determine the modal number. Karyotyping was performed according to the International System for Human Cytogenetic Nomenclature. DNA histogram analysis and chromosome analysis were carried out on the two cell lines at the 20th passage.

*DNA extraction and Southern blot hybridisation*

DNA from cultured cells was extracted using standard techniques (Sambrook *et al*., 1989). In brief, the cells were incubated in sodium dodecyl sulphate (SDS)-proteinase K and their DNA was extracted with phenol–chloroform–isoamyl alcohol. Cellular DNA (5 $\mu$g) was digested with an appropriate restriction enzyme and subjected to electrophoresis on agarose gels. DNA was transferred and then fixed to nylon filters. The DNA was then processed for Southern blotting hybridisation (Southern, 1975). The probes used were c-*myc* (Oncogene Science, Uniondale, NY, USA), *met*-D (Oncor, Gaithersburg, MD, USA), *met*-H (Oncor), v-*erb*B (Amersham, Tokyo, Japan) and c-*erb*B-2 (Amersham). In Southern blotting DNA was digested with restriction enzymes including *Eco*RI, *Sac*I and *Bgl*II (Boehringer Mannheim, Tokyo, Japan).

*Individual identification*

To demonstrate that OCUM-2M and OCUM-2D were derived from the same patient, we examined DNA polymorphisms of the two cell lines using variable number of tandem repeats (VNTR) DNA probes, which are valuable genetic markers for individual identification (Jeffreys *et al*., 1985; Nakamura *et al*., 1987). VNTR probes (pYNH24, TBQ7 and CMM101) were kindly supplied by the Japanese Cancer Research Resource Bank. VNTR probes pYNH24, TBQ7

**Figure 1** Morphological and ultrastructural findings of OCUM-2M cells and OCUM-2D cells. (**a**) Phase contrast photomicrograph of OCUM-2M cells. Most cells appear to be round, and form loose cell aggregates. (**b**) OCUM-2M cells exhibited various sized irregular nuclei in haematoxylin and eosin staining. (**c**) Electron micrograph of OCUM-2D cells showing large indented semiround nuclei and numerous microvilli on their surface, but few tight junctions. Many mitochondria were recognised in the cytoplasmic matrix. The endoplasmic reticulum and Golgi complex were not so well developed. Scale bars: (**a**) and (**b**), 30 $\mu$m; (**c**), 3 $\mu$m.

and CMM101 were located on chromosome 2, chromosome 10 and chromosome 14 respectively. DNA from cultured cells was extracted using standard techniques described above (Sambrook *et al.*, 1989). DNA was digested with *Hae*III and subjected to electrophoresis on agarose gels. DNA was transferred and then fixed to nylon filters. The DNA then was processed for Southern blotting hybridisation (Southern, 1975) and was probed with $^{32}$P-labelled pYNH24, TBQ7 and CMM101.

### *Production of tumour-associated antigens*

Carcinoembryonic antigen (CEA; Gold and Freedman, 1965), carbohydrate 19-9 (CA19-9; Koprowski *et al.*, 1979), cancer-associated antigen (SPan-1; Chung *et al.*, 1987), sialyl Tn antigen (STN; Kleldsen *et al.*, 1989), and sialyl Lewis X (SLX; Fukusi *et al.*, 1985) levels in the spent medium were measured by radioimmunoassay as follows. Cells ($1.0 \times 10^6$) were seeded into 100 mm Petri dishes (10 ml total volume) and cultured for 5 days; measurements were made of the supernatants. CEA was measured using CEA RIABEAD (Dainabot, Tokyo, Japan), CA19-9 using the Centcore CA19-9 radioimmunoassay, SPan-1 using SPan-1 RIABEAD (Dainabot), SLX using the Otuka SLX radioimmunoassay (Otsuka, Tokushima, Japan), and STN using the Otuka STN radioimmunoassay (Otsuka). As a control DMEM containing 10% FCS was used.

### *Expression of tumour-associated antigens on the cell surface*

Tumour-associated antigen expression on the cell surface was determined by flow cytometric analysis. The cells were prepared as a single cell suspension. Approximately $1.0 \times 10^6$ cells were treated individually in 1 ml of FACS buffer (phosphate-buffered saline with 0.1% sodium azide and 1% bovine serum albumin) with monoclonal antibody specific for CA19-9 (Dainabot), SPan-1 (Dainabot), CEA (Dainabot), SLX (Otsuka) and STN (Otsuka) at a final dilution of 1:100 for 60 min on ice, followed by washing twice (5 ml of FACS buffer) and labelling with fluorescein isothiocyanate-conjugated goat anti-mouse immunoglobulin (Tago, Burlingame, CA, USA) for 30 min on ice. The fluorescence of cells treated with all but the specific antibodies was used as control. After

**Table I** Biological characteristics of OCUM-2M and OCUM-2D cell lines

| *Biological properties* | *Cell lines* OCUM-2M | OCUM-2D |
|---|---|---|
| Growth mode | Suspension | Suspension |
| Doubling time (h) | 37.3 | 23.6 |
| Immunocytochemical stain | | |
| PAS | Positive | Positive |
| PAS with diastase pretreatment | Positive | Positive |
| Alcian blue | Negative | Negative |
| EGFR (fmol mg$^{-1}$ protein$^{-1}$) | 11.2 | 24.6 |
| Number of chromosomes | 70 | 70 |
| Ploidy pattern | Aneuploid | Aneuploid |
| DNA index | 1.592 | 1.820 |

**Table II** Tumorigenicity of OCUM-2M and OCUM-2D cells subcutaneously inoculated into nude mice

| *Cell line* | *No. of cancer cells* $1.0 \times 10^5$ | $1.0 \times 10^6$ | $5.0 \times 10^6$ |
|---|---|---|---|
| OCUM-2M | 0/5[a] | 1/5 | 2/10 |
| OCUM-2D | 0/5 | 3/5 | 10/10 |

[a]Number of mice bearing a tumour per total number of mice. Tumour-take was assessed 8 weeks after inoculation.

**Figure 2** The median tumour growth curves of the two cell lines, OCUM-2M (○) ($n = 2$) and OCUM-2D (●) ($n = 10$), grown as subcutaneous tumours in nude mice. The tumour size was expressed as the product of the largest tumour diameter by the shortest tumour diameter (mm²).

**Figure 3** DNA histograms of (a) normal lymphocytes, (b) OCUM-2M cells and (c) OCUM-2D cells.

two additional washes, the cells were analysed using a flow cytometer (Becton Dickinson).

*Enzyme-linked immunosorbent assay of epidermal growth factor receptor (EGFR)*

Enzyme-linked immunosorbent assay analysis of EGFR was performed using an EGFR tissue extract EIA kit (Triton Diagnostics, CA, USA). In brief, cell extracts were prepared from pelleted membrane-enriched homogenates. The cell extracts were prepared to a 0.1 mg $ml^{-1}$ final concentration and incubated with an anti-EGFR antibody. The extracts were then incubated with 3,3',5,5'-tetramethylbenzidine solution. The intensity of the colour formed by the enzymatic reaction was read with a spectrophotometer at 450 nm.

*Effect of epidermal growth factor (EGF) and transforming growth factor (TGF)-β1 on the growth of the cell lines and their morphology*

The effects of EGF (Gibco) and TGF-β1 (King Brewing, Kakogawa, Japan) on the growth and morphology of OCUM-2M and OCUM-2D cells at the 55th passage were examined. The tumour cells ($3.0 \times 10^4$) were plated in each well of 24-well dishes (Falcon) following the addition of EGF (0.1 ng $ml^{-1}$, 10 ng $ml^{-1}$) or TGF-β1 (0.1 ng $ml^{-1}$, 10 ng $ml^{-1}$) and incubated for 36 h, 72 h and 108 h. They were then observed by phase-contrast microscopy and counted. The doubling times were estimated from the growth curves.

*Invasion assay*

The difference in migratory capacity between the two cell lines was investigated with invasion assay by the method of Albini *et al.* (1987) with modifications. Invasion was measured by use of the Chemotaxicell chambers (Kubota) wiht a 12 μm porosity membrane filter and the upper surface of each filter was coated with 5 μg reconstituted basement membrane substance (Matrigel; Collaborative Research, Lexington, MA, USA) in cold DMEM per filter to form a matrix barrier. The chamber as the upper well was placed into a 24-well culture plate (Falcon) as a lower well. OCUM-2M and OCUM-2D cells were resuspended to a final concentration of $2 \times 10^4$ cells $ml^{-1}$ in DMEM with 10% FCS. Each tumour cell suspension (200 μl) was then added onto the Matrigel of the upper compartment of the chamber and incubated in the presence or absence of 10 ng $ml^{-1}$ TGF-β1 for 5 days at 37°C. The filters were fixed with methanol and stained with haematoxylin. The tumour cells on the upper surface of the filters were removed by wiping with cotton swabs. The cells which invaded through the Matrigel and the filter to the lower surface were counted manually under a microscope at a magnification of × 200. Four fields were counted for each assay. The mean of the four fields was calculated as the sample value. Each sample was assayed in triplicate and assays were repeated twice.

*Statistical analysis*

Data were analysed statistically using Student's *t*-test. A *P*-value less than 0.05 was considered statistically significant.

### Results

*Characteristics of the two cell lines*

Microscopic examination showed that the two cell lines OCUM-2M and OCUM-2D were similar in morphology. On phase-contrast microscopic examination, OCUM-2M cells (Figure 1a) and OCUM-2D cells were round and began to grow singly or in clusters in the culture medium. OCUM-2M cells (Figure 1b) and OCUM-2D cells both had various sized irregular nuclei by haematoxylin and eosin staining. On electron microscopic observation OCUM-2M cells and OCUM-2D cells (Figure 1c) were found to have many microvilli on their surfaces but few tight junctions. The biological characteristics of the two cell lines are summarised in Table I. The cytoplasm of each cell was stained with PAS with and without diastase pretreatment, but not with alcian blue. The cells were then noted to contain a mucinous substance in their cytoplasm.

The doubling times estimated from the growth curves of OCUM-2M and OCUM-2D at the 25th passage were 37.3 h and 23.6 h respectively. Tumour growth *in vivo* was observed in athymic BALB/c nude mice at the site of inoculation for 8 weeks. The tumorigenicity of OCUM-2D was greater than that of OCUM-2M. The inoculation of $5.0 \times 10^6$ OCUM-2D cells developed tumour formation with all treated mice (10/10), while the inoculation of the same number of OCUM-2M cells resulted in poor tumour formation (2/10; Table II). Figure 2 shows the median tumour growth curves of the two cell lines. The xenografted tumour produced by OCUM-2D cells grew more rapidly than that of OCUM-2M cells. Microscopic examination of the tumours revealed progressive growth of tumour cells, with numerous mitoses.

DNA histogram of both cell lines showed an aneuploid pattern. The DNA index of OCUM-2M was 1.592 and that of OCUM-2D was 1.820. The percentages of OCUM-2M and OCUM-2D cells in the $G_1$:S:$G_2$/M phases were 33.6:52.5:13.9% and 30.4:49.8:19.7% respectively (Figure 3). Chromosome analyses were carried out on both OCUM-2M and OCUM-2D cells at the 20th passage. Figure 4 shows the distribution of the number of chromosomes. The number of chromosomes of OCUM-2M cells ranged from 67 to 74 with a modal number of 70. The number of chromosomes of OCUM-2D cells ranged from 67 to 73 with a modal number of 70. Fifteen of 40 metaphase spreads examined were karyotyped. Figure 5 shows the major karyotypic features. The arrowheads indicate rearranged chromosomes. These chromosome abnormalities were present in most cells. The chromosome marker common to the two cell lines was a der(4)t(4;17)(q35;q11.2), which was present in

**Figure 4** Distribution of chromosome numbers. The OCUM-2M (**a**) and OCUM-2D (**b**) cells were analysed for chromosome number of 40 metaphase at the 20th passage.

all cells but duplicated in the metastasis. Other structural abnormalities of OCUM-2M were add(3)(p11), add(3)(q25) and del(10)(p11.2), and those of OCUM-2D were add(3)(p25), add(7)(p22), add(13)(p11), add(14)(q32), add(17)(p13) and add(20)(q13.3). OCUM-2M and OCUM-2D cells exhibited 5-fold and 3-fold amplification of the c-*myc* gene respectively (Figure 6a). Rearrangement of the c-*myc* gene was not detected (Figure 6b). No evidence of amplification was recognised in the other genes tested (*met*-D, *met*-H, v-*erb*B and c-*erb*B-2) (data not shown).

The same size alleles at pYNH24, TBQ7 and CMM101 probes were found in OCUM-2M and OCUM-2D cells (Figure 7), which indicates that OCUM-2M and OCUM-2D must be derived from the same patient.

The levels of tumour markers CA19-9, SPan-1, SLX and CEA in spent media of both cell lines were elevated compared with the control. The levels of these markers in OCUM-2M medium were higher than those in OCUM-2D medium. STN level was not elevated in the spent media (Table III). Figure 8 shows the levels of tumour marker expression on the cell surface. Level of expression on the cell surface was almost similar to that released into the medium except for STN. OCUM-2M cells strongly expressed CA19-9, SPan-1 and CEA on the cell surface compared with OCUM-2D cells. On the other hand, increase of STN expression was observed in OCUM-2D as compared with OCUM-2M.

### *Levels of EGFR expression*

Levels of EGFR expression on OCUM-2D cells were about twice those on OCUM-2M cells (Table I).

### *Effect of EGF and TGF-β1 on the growth of the cell lines and their morphology*

The growth of the OCUM-2D cells following the addition of 10 ng $ml^{-1}$ EGF was increased significantly by 45.7% relative to the untreated cells at 72 h after seeding, while that of

**Figure 5** G-banded karyotypes of OCUM-2M and OCUM-2D. (**a**) The representative karyotype of OCUM-2M was 70, XX, + 1, + 2, + add(3)(p11) × 2, add(3)(q25), der(4)t(4;17)(q35;q11.2), + 5, + 6, + 7, + 9, + del(10)(p11.2), + 11, + 12, + 14 × 2, + 15, + 16 × 3, + 19, + 20 × 2, + 22, + mar, + mar, + mar. (**b**) The representative karyotype of OCUM-2D was 70, XX, + 1, + 2, + add(3)(p25), + 4, der(4)t(4;17)(q35;q11.2) × 2, + 5, + 6, + add(7)(p22), + add(7)(p22), + 8, + 9, + 10, + 12 × 2, + add(13)(p11), + add(14)(q32), + 16 × 2, + add(17)(p13), + 19, + 20 × 2, + add(20)(q13.3), + 22, + mar. Arrowheads, breakpoints present.

OCUM-2M cells was increased by only 4.8% relative to the untreated cells. The growth of both OCUM-2M and OCUM-2D cells was decreased by 10 ng $ml^{-1}$ TGF-β1 (Figure 9). The doubling times estimated from the growth curve are summarised in Table IV. The doubling time of the OCUM-2D cells following the addition of 10 ng $ml^{-1}$ EGF was much shorter than that of the control cells. The doubling times of both OCUM-2M and OCUM-2D cells following the addition

**Figure 6** Amplification of the c-*myc* gene in OCUM-2M and OCUM-2D cells. (**a**) Southern blot analysis of DNA from placenta (lane 1), HL60 (lane 2), OCUM-2M (lane 3) and OCUM-2D (lane 4). The DNA (5 μg) was digested with restriction endonuclease *Eco*RI and analysed by hybridisation. Lane 1 represents a single copy, lane 2 represents four copies, lane 3 represents five copies and lane 4 represents three copies. (**b**) Southern blot analysis of DNA from OCUM-2M (lanes 5, 7 and 9), from OCUM-2D (lanes 6, 8 and 10) digested with three restriction enzymes including *Eco*RI, *Sac*I or *Bgl*II. No rearrangement of the c-*myc* gene was detected in either cell line.

**Figure 7** Individual identification using VNTR probes. DNA was digested with *Hae*III and probed with pYNH24 (**a**), TBQ7 (**b**) and CMM101 (**c**). Lane 1, OCUM-2M cells; lane 2, OCUM-2D cells. Arrowheads indicate the size of bands.

of 10 ng ml$^{-1}$ TGF-β1 were longer than those of the untreated cells, and were almost equal. On the other hand, the effects of TGF-β1 on cell morphology was different between the two cell lines. Attached and spreading cells were found following the addition of 10 ng ml$^{-1}$ TGF-β1 after 36 h culture. An increase in the number of attached and spreading cells was found following the addition of 10 ng ml$^{-1}$ TGF-β1 after 72 h culture, while most of the OCUM-2D cells without TGF-β1 were still round. After 108 h culture more of the cells with 10 ng ml$^{-1}$ TGF-β1 were attached and spreading more extensively and a few OCUM-2D cells without TGF-β1 began to be attached and to spread (Figure 10). OCUM-2M cells did not display this morphological change following the addition of TGF-β1 (data not shown).

**Table III** Levels of tumour markers in conditioned medium 5 days after seeding of 1 × 10$^5$ cells ml$^{-1}$

| | *CA19-9 (U ml$^{-1}$)* | *SPan-1 (U ml$^{-1}$)* | *CEA (ng ml$^{-1}$)* | *SLX (U ml$^{-1}$)* | *STN (U ml$^{-1}$)* |
|---|---|---|---|---|---|
| OCUM-2M | 6544 | 2299 | 18.9 | 52.2 | 12.5 |
| OCUM-2D | 168 | 89 | 4.8 | 20.6 | 12.5 |
| Control | 1 | 1 | 0.1 | 5 | 12.5 |

**Figure 8** Flow cytometric analysis of tumour marker surface expression by OCUM-2M (**a**) and OCUM-2D cells (**b**). Data are expressed as percentage of positive cells after subtraction of the control.

**Figure 9** Effect of EGF and TGF-β1 on the proliferation of OCUM-2M (**a**) and OCUM-2D (**b**) cells. Control (■); EGF 0.1 ng ml$^{-1}$ (●); EGF 10 ng ml$^{-1}$ (▲); TGF-β1 0.1 ng ml$^{-1}$ (◆); TGF-β1 10 ng ml$^{-1}$ (□). Cells were treated every 36 h. Results are means ± s.d. of four samples.

### *Migratory capacity of tumour cells*

The two cell lines were analysed for their invasive capacities using 12 μm porosity membrane filters coated with extracellular matrix composite, Matrigel. Many OCUM-2D cells invaded through the Matrigel and migrated to the lower side of the filters, while few OCUM-2M cells did. The migratory capacity of OCUM-2D cells was significantly increased following the addition of 10 ng $ml^{-1}$ TGF-β1 ($P<0.02$), while that of OCUM-2M cells was not (Figure 11).

### Discussion

Morphology of the two cell lines studied here were similar to each other and to scirrhous gastric cancer cell lines previously reported (Sekiguchi *et al.*, 1978; Yanagihara *et al.*, 1991, 1993). On phase-contrast microscopic examination cultured scirrhous gastric cancer cells have been reported to grow singly or in clusters (Motoyama *et al.*, 1986). The two cell lines we studied also exhibited these features. Our electron microscope studies demonstrated that cells in each of

**Table IV** The doubling time of OCUM-2M and OCUM-2D cells in the presence of EGF and TGF-β1

| Cell line[a] | Doubling time (h) None | 0.1 ng $ml^{-1}$ EGF | 10 ng $ml^{-1}$ EGF | 0.1 ng $ml^{-1}$ TGF-β1 | 10 ng $ml^{-1}$ TGF-β1 |
|---|---|---|---|---|---|
| OCUM-2M | 40.8 | 39.1 | 36.4 | 42.4 | 49.9 |
| OCUM-2D | 31.6 | 26.5 | 18.5 | 31.2 | 48.0 |

[a]OCUM-2M and OCUM-2D cells were examined at the 55th passage.

**Figure 10** The effect of TGF-β1 on the morphology of OCUM-2D cells. (**a**–**d**) No treatment. (**e**–**h**) In the presence of 10 ng $ml^{-1}$ TGF-β1. (**a** and **e**) Culture for 18 h. (**b** and **f**) Culture for 36 h. (**c** and **g**) Culture for 72 h. (**d** and **h**) Culture for 108 h. An increase in the number of attached and spreading cells was found following the addition of 10 ng $ml^{-1}$ TGF-β1 after 72 h culture, while most of OCUM-2D cells were still round without TGF-β1. Scale bar, 50 μm.

**Figure 11** Effect of TGF-$\beta$1 on the migratory capacity. Difference in migratory capacity between OCUM-2M and OCUM-2D cells was measured with invasion assay in the presence (■) or absence (□) of 10 ng ml$^{-1}$ TGF-$\beta$1. Many OCUM-2D cells invaded the Matrigel and migrated to the lower side of the filters, while few OCUM-2M cells did. The migratory capacity of OCUM-2D cells was significantly increased following the addition of 10 ng ml$^{-1}$ TGF-$\beta$1 ($P<0.02$), while that of OCUM-2M cells was not. Values are the mean of triplicate sample values. Bars, s.d.

the two lines have few attachments to each other. The cytoplasm of OCUM-2M and OCUM-2D cells exhibited positive PAS staining and it was also similar to that of most gastric cancer cell lines previously established.

On the other hand, a difference between OCUM-2M and OCUM-2D cells was recognised in the growth and invasive activities. OCUM-2D cells had higher proliferation and invasion than OCUM-2M cells. The viability of OCUM-2D cells, as evaluated by doubling time and tumorigenicity was higher than that of OCUM-2M cells. There have been reports that level of EGF and EGFR expression is correlated with depth of tumour invasion, frequency of metastasis and prognosis for human gastric carcinoma (Yasui *et al.*, 1988; Yoshida *et al.*, 1990). In our study, the level of EGFR expression on OCUM-2D cells was about twice that on OCUM-2M cells and EGF was much more effective in stimulating the growth of OCUM-2D cells than that of OCUM-2M cells. These findings suggest that high levels of growth activity may be needed for cancer cells to metastasise and that EGF might influence the growth activity.

In general, TGF-$\beta$1 has been reported to decrease the growth of most types of cancer cells. The growth of OCUM-2M and OCUM-2D cells was also decreased by addition of TGF-$\beta$1. It has been reported that the growth of some gastric cancer cell lines is not decreased by TGF-$\beta$1, owing to escape from the negative regulation of TGF-$\beta$1 at the receptor level (Ito *et al.*, 1992). However, there was no evidence for this escape in our two cell lines. On the other hand, migratory capacity of OCUM-2D cells was much greater than that of OCUM-2M cells. In our study, the motility of OCUM-2D cells was significantly stimulated following the addition of 10 ng ml$^{-1}$ TGF-$\beta$1, while that of OCUM-2M cells was not. Morphological changes of attached and spreading cells were recognised in OCUM-2D cells following treatment with 10 ng ml$^{-1}$ TGF-$\beta$1, but not in OCUM-2M cells. TGF-$\beta$1 has been thought to increase the invasive and metastatic potential of various tumour cells (Guirguits *et al.*, 1987; Mukai *et al.*, 1989; Mooradian *et al.*, 1992). Morphological changes (i.e. increased pseudopod formation) have been reported to be prominent features of active motile cells *in vitro* and of invasive tumour cells *in vivo* (Guirguits *et al.*, 1987; Mooradian *et al.*, 1992). The above findings suggest that high invasive capacity may play an important role in disseminated metastasis and might be partly affected by TGF-$\beta$1. The ability of OCUM-2D cells to change into attached and spreading cells following TGF-$\beta$1 may be associated with high invasive capacity. It has been reported that TGF-$\beta$1 is produced by most scirrhous gastric cancer cells and stromal cells such as fibroblasts (Yoshida *et al.*, 1989). We also observed production of TGF-$\beta$1 by OCUM-2D cells and gastric fibroblasts in immunoprecipitation studies (data not shown). It was thought that the invasive capacity of TGF-$\beta$1 may be influenced both in an autocrine and paracrine system. TGF-$\beta$1 produced from OCUM-2D cells was approximately twice that from OCUM-2M cells. In addition, production of urokinase-type plasminogen activator (u-PA), which activates the latent TGF-$\beta$1, was approximately six times greater in OCUM-2D cells than in OCUM-2M cells by enzyme-linked immunosorbent assay (data not shown). These findings may contribute towards the differential invasive capacities of the two cell lines.

It has been reported that most gastric cancer cells produce some tumour-associated antigens (Motoyama *et al.*, 1986). OCUM-2M and OCUM-2D cells also produced the tumour-associated antigens CA19-9, CEA, SPan-1 and SLX. Tumour-associated antigen expression on the cell surface was also recognised in each cell line by flow cytometric analysis. Level of expression on the cell surface was also similar to that released into the medium except for STN. Recently, tumour-associated antigens have been reported to function as adhesion molecules. In some studies, it has been found that overexpression of tumour-associated antigens, CA19-9 and SLX, was responsible for adhesion to human umbilical vein endothelial cells in liver metastatic lesions (Matsushita *et al.*, 1990; Takada *et al.*, 1993). In these studies, it was reported that the adhesion of colon and pancreas cancer cell lines was almost exclusively dependent on CA19-9 and that the adhesion of lung and liver cancer cells was dependent on SLX. Another study has reported that surface mucins, which contain carbohydrate antigens such as CA19-9 and SPan-1, inhibited pancreatic cancer cell adhesion to the substratum (Sawada *et al.*, 1993). The cell surface expression levels of tumour-associated antigens CA19-9, SPan-1 and CEA were lower in OCUM-2D cells than those in OCUM-2M cells. Reduction of expression of carbohydrate antigens might be beneficial for adhesion to the substratum of the peritoneum and pleura in disseminated metastasis. CEA is reported to be a cell-to-cell adhesion molecule (Benchimol *et al.*, 1989). Reduced expression of CEA may help cells to separate from each other and this separation may enable cancer cells to escape from primary tumour and to invade. The above findings suggest that reduction in the expression of tumour-associated antigens, including CA19-9, SPan-1 and CEA, might be necessary for disseminated metastases, which are distinct from liver metastasis, in scirrhous gastric carcinoma.

It has recently been reported that the amplification of specific oncogenes is associated with tumour stage and the prognosis of cancer (Tujino *et al.*, 1990). The c-*myc* oncogene has been reported to play an important role in cell proliferation (Ranzani *et al.*, 1990). Some studies have reported that c-myc protein accumulation or DNA amplification is related to the clinical stage and prognosis of gastric cancer, and have suggested that the c-*myc* oncogene plays an important role in cancer progression (Ranzani *et al.*, 1990; Ninomiya *et al.*, 1991). In various gastric cancer cell lines, the c-*myc* oncogene has been reported to be amplified (Allum *et al.*, 1987; Ninomiya *et al.*, 1991). Both the OCUM-2M and OCUM-2D cell lines also exhibited c-*myc* amplification, however c-*myc* amplification in OCUM-2M cells was greater than that in the OCUM-2D cells. This finding suggests that amplification of the c-*myc* oncogene might have occurred at an early stage and might not play an important role in the process of disseminated metastases. In a report, however, metastatic lesions were found to have higher levels of c-*myc* mRNA than primary lesions (Kelly *et al*., 1983). The rate of c-*myc* gene transcription has been reported to be affected by factors such as EGF and TGF-$\beta$1 (Coffey *et al*., 1988; Pietenpol *et al*., 1990*a,b*). It will therefore be important in future studies to determine the effects of EGF and TGF-$\beta$1 on levels of c-*myc* mRNA of OCUM-2M and OCUM-2D cells.

In conclusion, we have established two new cell lines, OCUM-2M and OCUM-2D from a primary tumour and a metastatic lesion respectively from a patient with scirrhous gastric carcinoma. OCUM-2D cells demonstrated higher proliferation and invasion than OCUM-2M cells. It was suggested that these activities may play an important role in disseminated metastasis in scirrhous gastric carcinoma and might be partly affected by EGF and TGF-$\beta$1.

**Abbreviations**

DMEM, Dulbecco's modified Eagle medium; FCS, fetal calf serum; EGF, epidermal growth factor; EGFR, epidermal growth factor receptor; TGF-$\beta$1, tranforming growth factor-$\beta$1; PAS, periodic acid-Schiff; VNTR, variable number of tandem repeats; CEA, carcinoembryonic antigen; CA19-9, carbohydrate 19-9; SPan-1, cancer-associated antigen SPan-1; SLX, sialyl Lewis X-i; STN, sialyl Tn antigen; u-PA, urokinase-type plasminogen activator.

**References**

ALBINI A, IWAMOTO Y, KLEIMAN KH, MARTIN RG, AARONSON SA, KOZLOWSKI JM AND McEWAN RN. (1987). A rapid *in vitro* assay for quantitating the invasive potential of tumor cells. *Cancer Res*., **47**, 3239-3245.

ALLUM WH, NEWBOLD KM, MACDONALD F, RUSELL B AND STOCKES H. (1987). Evaluation of p62$^{c\text{-}myc}$ in benign and malignant gastric epithelia. *Br. J. Cancer*, **56**, 785-786.

BENCHIMOL S, FUKS A, JOTHY S, BEAUCHEMIN N, SHIROTA K AND STANNER CP. (1989). Carcinoembryonic antigen, a human tumor marker, functions as an intercellular adhesion molecule. *Cell*, **57**, 327-334.

CHUNG YS, HO JJL, KIM YS, TANAKA H, NAKATA B, HIURA A, MOTOYOSHI H, SATAKE K AND UMEYAMA K. (1987). The detection of human pancreatic cancer-associated antigen in the serum of cancer patients. *Cancer*, **60**, 1636-1643.

COFFEY Jr JR, BASCOM CC, SIPES NJ, GRAVES-DEAL R, WEISSMAN BE AND MOSES HL. (1988). Selective inhibition of growth related gene expression in murine keratinocytes by transforming growth factor $\beta$. *Mol. Cell. Biol*., **8**, 3088-3093.

FUKUSI Y, KANNAGI R AND HAKOMORI S. (1985). Location and distribution of defucogangrioside in normal and tumor tissues defined by its monoclonal antibody FH6. *Cancer Res*., **45**, 3711-3717.

GOLD P AND FREEDMAN SO. (1965). Demonstration of tumor-specific antigens in human colon carcinoma by immunological tolerance and absorption techniques. *J. Exp. Med*., **121**, 439-471.

GUIRGUITS R, MARGUIES I, TARABOLETTI G, SCHIFFMANN E AND LIOTTA L. (1987). Cytokine-induced pseudopodial protrusion is coupled to tumor cell migration. *Nature*, **329**, 261-263.

ITO M, YASUI W, KYO E, YOKOZAKI H, NAKAYAMA H, ITO H AND TAHARA E. (1992). Growth inhibition of transforming growth factor $\beta$ on human gastric carcinoma cells: receptor and post-receptor signaling. *Cancer Res*., **52**, 295-300.

JEFFREYS AJ, WILSON V AND THEIN SL. (1985). Individual-specific 'fingerprints' of human DNA. *Nature*, **316**, 76-79.

KELLY K, COCHRAN BH, STILE CD AND LEDE P. (1983). Cell specific regulation of the c-*myc* gene by lymphocyte mitogens and platelet-derived growth factor. *Cell*, **35**, 603-610.

KIYASU Y, KANESHIMA S AND KOGA S. (1981). Morphogenesis of peritoneal metastasis in human gastric cancer. *Cancer Res*., **41**, 1236-1239.

KLELDSEN T, CLAUSEN H, HIROHASHI S, OGAWA T, IIJIMA H AND HAKOMORI S. (1989). Preparation and characterization of monoclonal antibodies directed to the tumor-associated O-linked sialosyl-2→6 α-N-Acetylgalactosaminyl (sialosyl-Tn) epitope. *Cancer Res*., **48**, 2214-2220.

KOPROWSKI H, STEPLEWSKI Z AND MITCHELL L. (1979). Colorectal carcinoma antigens detected by hybridoma antibodies. *Somat. Cell Genet*., **5**, 957-972.

MATSUSHITA Y, CLEARY KR, OTA DM, HOFF SD AND IRIMURA T. (1990). Sialyl-dimeric Lewis-X antigen expressed on mucin-like glycoproteins in colorectal cancer metastasis. *Lab. Invest*., **63**, 780-791.

MOORADIAN LD, McCARTHY BJ, KOMANDURI VK AND FURCHT TL. (1992). Effects of transforming growth factor-$\beta$1 on human pulmonary adenocarcinoma cell adhesion, motility, and invasion *in vitro*. *J. Natl Cancer Inst*., **84**, 523-527.

MOTOYAMA T, HOJO H AND WATANABE H. (1986). Comparison of seven cell lines derived from human gastric carcinomas. *Acta Pathol. Jpn*., **36**, 65-72.

MUKAI M, SHINKAI K, KOMATU K AND AKEDO H. (1989). Potentiation of invasive capacity of rat ascites hepatoma cells by tranforming growth factor-$\beta$. *Jpn. J. Cancer Res*., **80**, 107-110.

NAKAMURA Y, LEPPERT M, O'CONNELL P, WOFF R, HOLM T, CULVER M, MARTIN S, FUJIMOTO E, HOFF M, KUMLIN E AND WHITE R. (1987). Variable number of tandem repeat (VNTR) markers for human gene mapping. *Science*, **235**, 1616-1622.

NINOMIYA I, YONEMURA Y, MATUMOTO H, SUGIYAMA K, MIWA K, MIYAZAKI I AND SHUKU H. (1991). Expression of c-*myc* gene product in gastric carcinoma. *Oncology*, **48**, 149-153.

PIETENPOL JA, HOLT JT, STEIN RW AND MOSES LH. (1990*a*). Transforming growth factor $\beta$1 suppression of c-*myc* gene transcription: role in inhibition of keratinocyte proliferation. *Proc. Natl Acad. Sci*., **87**, 3758-3762.

PIETENPOL JA, STEIN RW, MORAN E, YACIUK P, SHLEGEL R, LYONS RM, PITTELKOW MR, MUNGER K, HOWLEY PM AND MOSES HL. (1990*b*). TGF-$\beta$1 inhibition of c-*myc* transcription and growth in keratinocytes is abrogated by viral transforming proteins with pRB binding domains. *Cell*, **61**, 777-785.

RANZANI GN, PELLEGETA NS, PREVIDERE C, SARAGONI A, VIO A, MALTONI M AND AMADORI D. (1990). Heterogenous protooncogene amplification correlates with tumor progression and presence of metastases in gastric cancer patients. *Cancer Res*., **50**, 7811-7814.

SAMBROOK J, FRITCH EF AND MANIATIS T. (1989). *Molecular Cloning: A Laboratory Manual*, 2nd edn., pp.916-919. Cold Spring Harbor Laboratory Press: Cold Spring Harbor, New York.

SAWADA T, HO JJJ, SOGABE T, YOON WH, CHUNG YS, SOWA M AND KIM YS. (1993). Biphasic effect of cell surface sialic acids on pancreatic cancer cell adhesiveness. *Biochem. Biophys. Res. Commun*., **195**, 1096-1103.

SEABRIGHT M. (1971). A rapid banding technique for human chromosomes. *Lancet*, **2**, 971-972.

SEKIGUCHI M, SAKAKIBARA K AND FUJII G. (1978). Establishment of cultured cell lines a human gastric carcinoma. *Jpn. J. Exp. Med*., **48**, 61-69.

SOUTHERN EM. (1975). Detection of specific sequences among DNA fragments separated by gel electrophoresis. *J. Mol. Biol*., **98**, 503-517.

TAHARA E. (1990). Growth factors and oncogenes in human gastrointestinal carcinomas. *J. Cancer Res. Clin. Oncol*., **116**, 121-131.

TAKADA A, OHMORI K, YONEDA T, TSUYUOKA K, HASEGAWA A, KISO M AND KANNAGI R. (1993). Contribution of carbohydrate antigens sialyl Lewis A and sialyl Lewis X to adhesion of human cancer cells to vascular endothelium. *Cancer Res*., **53**, 354-361.

TERASHIMA M, IKEDA K, MAEAWA C, KAWAMURA H, NIITU Y, SATOH M AND SAITO K. (1991). Establishment of an α-fetoprotein-producing gastric cancer cell line in serum-free media. *Jpn. J. Cancer Res*., **82**, 883-885.

TUJINO T, YOSHIDA K, NAKYAMA H, ITO H AND TAHARA E. (1990). Alterations of oncogenes in metastatic tumors of human gastric carcinomas. *Br. J. Cancer*, **62**, 226-230.

YANAGIHARA K, SEYAMA T, TSUMURAYA M, KAMADA N AND YOKORO K. (1991). Establishment and characterization of human signet ring cell gastric carcinoma cell lines with amplification of the c-*myc* oncogene. *Cancer Res*., **51**, 381-386.

YANAGIHARA K, KAMADA N, TSUMURAYA M AND AMANO F. (1993). Establishment and characterization of a human gastric scirrhous carcinoma cell line in serum-free chemically defined medium. *Int. J. Cancer*, **54**, 200-207.

YASUI W, SUMIYOSHI K, HATA J, KAMEDA T, OCHIAI A, ITO H AND TAHARA E. (1988). Expression of epidermal growth factor receptor in human gastric and colonic carcinomas. *Cancer Res*., **48**, 137-141.

YOSHIDA K, YOKOZAKI H, NIIMOTO M, ITO H, ITO M AND TAHARA E. (1989). Expression of TGF-β1 and procollagen type I and type III in human gastric carcinomas. *Int. J. Cancer*, **44**, 394-398.

YOSHIDA K, KYO E, TUJINO T, SANO T, NIMOTO M AND TAHARA E. (1990). Expression of epidermal growth factor, transforming growth factor-α and their receptor genes in human gastric carcinomas: implication for autocrine growth. *Jpn. J. Cancer Res.*, **81**, 43-51.